# Incidents of sudden death during restraint of agitated individuals in Sweden between 1992 and 2024

**DOI:** 10.1111/1556-4029.70237

**Published:** 2025-11-25

**Authors:** Alexander Tyr, Erik Lindeman, Fredrik Tamsen, Ingemar Thiblin, Brita Zilg

**Affiliations:** ^1^ Swedish National Board of Forensic Medicine Stockholm Solna Sweden; ^2^ Department of Laboratory Medicine Karolinska Institutet Huddinge Sweden; ^3^ Swedish Poisons Information Centre Solna Sweden; ^4^ Swedish National Board of Forensic Medicine Uppsala Uppsala Sweden; ^5^ Department of Surgical Sciences Akademiska Sjukhuset, Uppsala University Uppsala Sweden; ^6^ Department of Oncology‐Pathology Karolinska Institutet Stockholm Sweden

**Keywords:** acidosis, arrest‐related deaths, arterial blood gas, autopsy, excited delirium, forensic pathology, hyperactive delirium, medico‐legal death investigations, positional asphyxia, prone restraint, restraint asphyxia, sudden cardiac death

## Abstract

Restraint‐related sudden deaths in agitated individuals raise complex questions at the intersection of medicine and law. Hyperactive delirium with extreme agitation as well as positional asphyxia due to restraint have been proposed to account for these deaths. However, the exact physiological mechanisms responsible and to what extent restraint contributes to the lethal outcome remain debated. In this nationwide, 32‐year retrospective study between 1992 and 2024, we examined circumstances surrounding sudden deaths during restraint of agitated individuals in Sweden. A total of 52 cases were identified, with an average of 0.17 deaths per million inhabitants annually. Ninety percent of cases involved prone restraint and 69% showed evidence of stimulant use. In 15 cases from 2005 onward, peri‐arrest arterial blood gas data revealed profound metabolic and respiratory acidosis, with a mean blood pH of 6.52 (range: 6.30–6.95; median: 6.50), mean lactate concentration of 26.3 mmol/L (range: 8.6–41.0; median: 30), and mean pCO_2_ of 14.8 kPa (range: 6.4–22.3; median: 15.3). Based on these findings, we propose a two‐phase pathophysiological model of restraint‐related cardiac arrest. The initial “priming phase” involves extreme physical exertion, creating a critically acidotic state that requires full respiratory and cardiovascular function to maintain homeostasis. If the “priming phase” is followed by restraint that restricts ventilatory function and hampers venous return, e.g., restraint in the prone position, an unstable “tipping phase” is initiated, that may culminate in cardiac arrest. This model builds on previous hypotheses and emphasizes the potentially lethal consequences of inhibiting ventilatory function in acutely agitated individuals.

**Highlights**

- Sudden death during restraint of agitated individuals remains medically and legally contentious.
- Nationwide 32‐year study identified 52 fatalities in Sweden.
- Prone restraint is documented in 90% of cases.
- Peri‐arrest arterial blood gases demonstrate profound combined metabolic–respiratory acidosis.
- Propose a two‐phase model to describe restraint‐related cardiac arrest in agitated individuals.

## 1 INTRODUCTION

Sudden death of agitated individuals during restraint by law enforcement officers is a rare but recurring phenomenon worldwide [1]. In Sweden, forensic pathologists assess the cause and manner of death, providing essential input to the court that is legally responsible for determining whether a restraint‐related death is the result of a criminal offense. This distinction carries significant legal implications in determining liability for the outcome of the altercation. Inadequate understanding or failure to clearly explain the physiological mechanisms underlying restraint‐related deaths may invoke extra‐legal ramifications. Public discourse surrounding such incidents is often contentious, but may become less so if there is greater agreement on the causes of death within the medico‐legal community. More importantly, a realistic understanding of the physiological risks associated with restraining severely agitated individuals is a precondition for the development of protocols to mitigate harm when such individuals are handled by law enforcement and healthcare professionals.

There is a lack of consensus regarding the underlying pathophysiological mechanisms leading to death in the English‐language literature to date. The classification of agitation‐related deaths as excited delirium (ExD) has been subject to criticism [2, 3, 4]. Several medical organizations, including the American Psychiatric Association (APA) and the World Health Organization (WHO) have never acknowledged the diagnosis, and the National Association of Medical Examiners (NAME), the College of American Pathologists (CAP), as well as the American College of Emergency Physicians (ACEP), have recently withdrawn their classification of the term. In Sweden, ExD is often referred to as “agitated delirium” within medico‐legal discussions, a term essentially synonymous with ExD and equally controversial. There exists a general consensus within the international community that a state of acute delirium and agitation increases the risk of restraint‐related death, and several associations have agreed on “hyperactive delirium with extreme agitation” as a preferred term [5, 6]. Nevertheless, agitation and hyperactivity on their own are considered insufficient to explain the cause of death and have been deemed impermissible in several court systems in the United States [2, 3]. Other causes of death that have been proposed are traumatic or positional asphyxia, which both posit similar pathological mechanisms of death related to a mechanical prevention of breathing [7]. However, the explanatory framework is difficult to reconcile with the sudden onset of cardiac arrest observed in individuals who were verbally responsive moments before, and it fails to account for the high incidence of unsuccessful resuscitation in restraint‐related deaths, even when promptly initiated [8].

Toxicological screening frequently detects substances of abuse in cases of restraint‐related deaths [9, 10, 11, 12]. Internationally, cocaine and methamphetamine appear to be the most common stimulants identified, while amphetamine has been found to dominate in Sweden [13]. However, deaths where no substances are detected are not uncommon, proving that intoxication is not necessary for death to occur. The lack of a clear understanding of the mechanisms leading to death is evident in Swedish medico‐legal death certificates, where the cause of death given in the original autopsy report varies between exhaustion, asphyxia due to chest compression, agitated delirium, drug intoxication, and cardiovascular‐related disease, despite strikingly similar peri‐mortal circumstances. Evidently, there exists a need for further research to enhance our understanding of common factors associated with the restraint of agitated and delirious individuals.

Numerous articles have addressed various challenges associated with the subject, ranging from reviews, case reports, and commentaries [4, 14, 15, 16, 17, 18, 19, 20, 21, 22]. However, studies involving larger series of restraint‐related deaths are lacking. In this national retrospective study of sudden deaths during restraint in Sweden between 1992 and 2024, we aim to identify patterns and contributing factors to enhance our understanding of the physiological mechanisms involved. By comprehensively analyzing case data over a 32‐year period, this study seeks to characterize the circumstances surrounding these incidents, evaluate key physiological parameters, and assess potential temporal trends. The findings will provide insights into deaths occurring during restraint by law enforcement, healthcare professionals, and bystanders, contributing to a broader understanding of risk factors and underlying mechanisms. The study also aims to enhance consistency in forensic assessments and inform the development of standardized guidelines for forensic practitioners, as well as promoting the safe management of agitated individuals to prevent future fatalities.

## 2 METHODS

In Sweden, deaths deemed as unexpected or potentially unnatural by the Police Authority are subject to medico‐legal autopsy and toxicological screening, both carried out by the Swedish National Board of Forensic Medicine (NBFM). Findings are systematically recorded by the attending forensic pathologist into a centralized national database, which includes demographic information (e.g., age and sex), circumstances of death, as well as the certified cause and manner of death. All cases involving deaths in connection with arrest and restraint by various law enforcement officers, healthcare personnel, or civilians between January 1, 1992, and December 31, 2024, were identified by free‐text searches in the NBFM database. Only deaths involving restraint were included, while fatalities resulting from shootings, car chases, or other forms of violence were excluded. Autopsy and police reports as well as medical records from emergency clinics were reviewed to extract relevant variables.

Data management and analysis were carried out using Microsoft Excel 2019 (version 1808) and arranged into three groups: population demographics and potential risk factors, restraint characteristics, and medico‐legal findings. For population demographics we extracted information regarding sex, age, and year of death. For potential risk factors, we assessed body mass index (BMI), categorized as either underweight (<18.5 kg/m^2^), normal weight (18.5–24.9 kg/m^2^), overweight (25–29.9 kg/m^2^), or obese (>30 kg/m^2^), and examined if there was a known history of mental illness.

In terms of restraint characteristics, details were sourced regarding the restraining authority (categorized as either police, security guard, prison guard, nursing staff, or civilian), the restraint type; defined as handcuffs (hands are cuffed together behind the back), leg cuffs, body cuff (handcuffs attached to hip belt, arms in a neutral side position), hogtie (hand cuffs and legcuffs are attached behind the back), or mechanical restraint (immobilization of an individual in a hospital bed by means of straps). Body position during restraint was considered as either prone (chest, abdomen, and hips toward the floor), supine or side, and information was gathered regarding the use of pressure applied or a hold/lock grip. The administration of medication during restraint was also considered, alongside whether cardiopulmonary resuscitation (CPR) had been performed. Although conductive energy devices were only recently introduced to Swedish police in 2022 and remain illegal for the general public, their use was documented, in addition to other relevant factors including the use of oleoresin capsicum (OC) spray, batons, or the involvement of police dogs that may increase stress and aggression.

Medico‐legal findings included autopsy records, toxicological analysis (divided into alcohol and stimulant concentration, where feasible) as well as the assessed cause of death. Toxicological analysis was performed using gas chromatography as described previously [23, 24, 25, 26]. Only significant/serious or life‐threatening injuries were included and bruises, abrasions and injuries related to CPR were excluded. Perimortem arterial blood gas (ABG) analyses performed at hospital emergency departments were also collected from 2005 to 2024, and consisted of pH, pO_2_ (kPa), pCO_2_ (kPa), lactate, and base excess concentrations.

### 2.1 Ethical considerations

This national study involves no identifiable patient data and therefore no ethical restraints by Swedish law were applicable, as stated by the Swedish Ethical Review Board (decision date April 2, 2025) (Dnr 2025‐01698‐01).

## 3 RESULTS

### 3.1 Population demographics and potential risk factors

**FIGURE 1:**
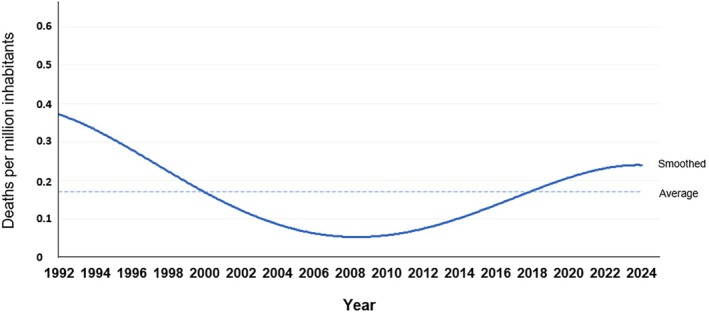
Incidence of sudden death during restraint of agitated individuals in Sweden (1992–2024). Temporally resolved smoothed (solid blue line) and period‐averaged (dashed blue line) data. During the investigated period, 52 restraint‐related deaths were identified, averaging 0.17 deaths per million inhabitants per year.

We identified 52 cases of sudden death during restraint, 50 (96%) males and 2 (4%) females with a mean age of 37 years (range 20–61 years) (Table 1). The age distribution was 13 (25%) individuals between ages 20 and 29, 21 (40%) between 30 and 39 and the remaining 18 (35%) over 40 years old. The number of restraint‐related deaths varied over the study period, with fluctuations across decades (Figure 1). In the 1990s, an average of 2.5 cases (0.28 per million inhabitants) per year was observed, with the number decreasing in the 2000s and 2010s, averaging 1 case (0.1 per million inhabitants) per year. However, from 2020 onward, the number of cases increased, with an average of 2.4 cases (0.23 per million inhabitants) per year.

**TABLE 1:** Population demographics and potential risk factors.

	*n*	%
**Sex**		
Male	50	96%
Female	2	4%
**Age**		
20–29	13	25%
30–39	21	40%
40–61	18	35%
**BMI** ^a^		
Underweight	1	2%
Normal weight	12	23%
Overweight	20	38%
Obese	15	29%
No data	4	8%
**History of mental illness**		
Yes	23	44%
No	29	56%

^a^ BMI presented as either underweight (<18.5 kg/m^2^), normal weight (18.5–24.9 kg/m^2^), overweight (25–29.9 kg/m^2^), or obese (>30 kg/m^2^).

According to BMI classification, one participant (2%) was underweight, while 12 (23%) had a normal weight. Twenty individuals (38%) were classified as overweight, and 15 (29%) were categorized as obese. BMI data were unavailable for four participants (8%). A history of diagnosed psychiatric conditions was reported in 23 participants (44%), including disorders of mood, psychosis, neuropsychiatric, and personality disorder.

### 3.2 Restraint characteristics

A summary of the restraint characteristics is shown in Table 2. In 31 cases (60%), the police authority conducted the restraint; six cases (12%) occurred with security guards, four cases (8%) occurred with nursing staff, and four (8%) with prison guards. The remaining cases were a combination of police with security guards (2 cases, 4%), police with nursing staff (2 cases, 4%), and with civilians (4 cases, 8%). Handcuffs were used in 36 cases (69%) and in 17 (47%) cases together with leg cuffs. Leg cuffs were never used without handcuffs, and hogtie restraints were identified in four cases (8%). A mechanical restraint was applied in 3 cases (6%), and in a single case (2%), a body cuff was used. In 14 cases (27%), no cuffing was reported. In terms of main body position, the majority of cases were placed prone (47 cases, 90,%); one (2%) was reported to be positioned in supine, two on their side (4%), one (2%) in a sitting position and in one case (2%), the body position during arrest was unknown.

**TABLE 2:** Restraint characteristics.

Case number	Arresting body	Restraint type	Main body position	Applied pressure	CPR attempted at scene	Other relevant information
1	Police	Handcuffs, legcuffs (Hogtie)	Prone	Back, buttocks	No	–
2	Police	Handcuffs, legcuffs (Hogtie)	Prone	None	Yes	–
3	Police	None	Prone	Back	Yes	–
4	Police	Handcuffs, legcuffs	Prone	None	Yes	–
5	Prison guard	Handcuffs, legcuffs (Hogtie)	Prone	Neck, back	No	–
6	Police	Handcuffs	Prone	Back	Yes	–
7	Police	Handcuffs, legcuffs	Prone	None	Yes	–
8	Nursing staff	None	Prone	None	Yes	–
9	Nursing staff	None	Prone	None	Yes	–
10	Security guard	None	Prone	Choke hold, neck, back	Yes	–
11	Police, nursing staff	Handcuffs	Prone	Back	Yes	Administration of depressant medication
12	Police	Handcuffs	Prone	Neck, back	No	Police dog
13	Civilian, police	Handcuffs, legcuffs	Prone	Back, buttocks	Yes	–
14	Security guard	None	Prone	Choke hold, neck, back, buttocks	Yes	–
15	Security guard	None	Prone	Neck, back, buttocks	Yes	–
16	Security guard	Handcuffs	Prone	Back	No	Baton
17	Police	Handcuffs	Prone	Back, buttocks	Yes	–
18	Police	Mechanical restraint	Prone	Neck	Yes	–
19	Police	Handcuffs	Prone	Back, buttocks	Yes	–
20	Police	Handcuffs	Prone	None	Yes	Baton
21	Prison guard	None	Prone	Choke hold, neck, back, buttocks	Yes	–
22	Police	Handcuffs Mechanical restraint	Prone	Back	Yes	–
23	Police	Handcuffs	Prone	None	Yes	–
24	Police, nursing staff	Handcuffs	Supine	None	Yes	–
25	Police	None	Prone	Back	Yes	OC spray
26	Civilian	None	Side	Choke hold	Yes	–
27	Nursing staff	None	Prone	Buttocks	Yes	–
28	Nursing staff	Handcuffs	Prone	Back, buttocks	Yes	–
29	Police	Handcuffs	Prone	Neck, back, buttocks	Yes	OC spray, baton
30	Police	Handcuffs, legcuffs (Hogtie)	Side	None	Yes	–
31	Police	Handcuffs, legcuffs	Prone	None	Yes	OC spray
32	Security guard, police	Handcuffs, legcuffs	Prone	Neck, back	Yes	OC spray
33	Police	Handcuffs, legcuffs	Prone	Back, buttocks	Yes	OC spray, baton
34	Prison guard	Bodycuff	Upright sitting	Choke hold, neck	Yes	–
35	Police	Handcuffs, legcuffs Mechanical restraint	Prone	None	No	Spit hood
36	Police	Handcuffs, legcuffs	Prone	None	Yes	OC spray
37	Civilian, police	None	Prone	Choke hold, neck, back, buttocks	Yes	–
38	Civilian, police	None	Prone	Back	Yes	–
39	Police	Handcuffs	Unknown	Unknown	Yes	–
40	Police	Handcuffs	Prone	Back, buttocks	Yes	–
41	Police	Handcuffs	Prone	Back	Yes	–
42	Police	Handcuffs	Prone	Back, buttocks	Yes	OC spray
43	Police	Handcuffs, legcuffs	Prone	Back	Yes	OC spray, spit hood
44	Police	Handcuffs	Prone	None	Yes	–
45	Security guard	Handcuffs	Prone	Choke hold, back	Yes	–
46	Police	Handcuffs, legcuffs	Prone	Back	Yes	OC spray, spit hood
47	Police	None	Prone	None	Yes	OC spray
48	Civilian, police	None	Prone	Back	Yes	–
49	Police	Handcuffs, legcuffs	Prone	None	Yes	–
50	Police	Handcuffs	Prone	Choke hold	Yes	OC spray
51	Security guard, police	Handcuffs, legcuffs	Prone	None	Yes	–
52	Police	Handcuffs, legcuffs	Prone	None	Yes	OC spray

Physical pressure and/or manual holds were reported in 34 cases (65%). Among these, force was applied to the back in 29 cases (85%), to the neck in 11 cases (33%), to the buttocks in 14 cases (41%), and a choke hold was reported in 7 cases (22%). Sedative medication (diazepam) was administered during restraint in only one case (2%). During the physical altercation, OC spray was used in 12 cases (23%), a baton in 4 cases (8%), and a police dog in a single case (2%). A spit hood was applied in 4 cases (8%). No cases included the use of conductive energy devices. CPR was performed in 47 cases (90%).

### 3.3 Medico‐legal findings

Perimortem ABG values were available sporadically in the material, with 15 samples recorded from 2005 onward (Table 3). Of these, pH and lactate levels were documented in all 15 cases (100%), pCO_2_ in 13 cases (87%), pO_2_ in 8 cases (53%), and base excess in 10 cases (67%). In one additional case, “acidosis” was noted without detailed ABG parameters. ABGs were collected, on average, 53 mins following struggle onset (median: 51.5 mins; range: 29–81 mins). The mean blood pH was 6.52, with values ranging from 6.30 to 6.95 (median pH 6.50). The mean lactate concentration was 26.3 mmol/L (range: 8.6–41.0 mmol/L) with a median of 30 mmol/L. The mean pCO_2_ was 14.8 kPa (range: 6.4–22.3 kPa) with a median of 15.3 kPa.

**TABLE 3:** Results from blood gas analysis, available from 2005.

Case number	Time from fight/restraint onset to blood sample (h:mm)	pH Normal range 7.35–7.45	Lactate (mmol/L) Normal range 0.5–2.2	pCO_2_		pO_2_		Base excess mEq/L Normal range −2 to +2
				kPa Normal range 4.7–6.0	mmHg Normal range 35–45	kPa Normal range 10.5 –13.5	mmHg Normal range 80–100	
26	1:21	6.87	–	–	–	–	–	−16
27	Blood gas analysis not performed.							
28	Blood gas analysis not performed.							
29	0:48	6.35	41	17	127.7	21	157	−29
30	Blood gas analysis not performed.							
31	1:00	<6.5	38	17	127.7	–	–	–
32	1:04	6.70	29	6.4	48	–	–	−30
33	0:55	6.63	26	13.9	104.3	5.97	44.78	−24
34	Individual parameters not available, record states patient was acidotic.							
35	Blood gas analysis not performed.							
36	Blood gas analysis not performed.							
37	Blood gas analysis not performed.							
38	Blood gas analysis not performed.							
39	0:35	6.80	15	15.3	114.8	–		−21
40	0:44	6.40	30	15.7	117.8	–		−15
41	0:44	6.95	8.6	11.2	84.8	2.2	16.5	−14
42	1:03	6.3	31	21.8	163.5	3.4	25.5	−39
43	Blood gas analysis not performed.							
44	1:05	6.47	>30	16	120	–	–	–
45	1:06	6.40	30	15.1	113.3	5.4	40.5	–
46	Blood gas analysis not performed.							
47	0:47	6.64	14.5	13.7	102.8	6.2	46.5	−26
48	Blood gas analysis not performed.							
49	Blood gas analysis not performed.							
50	0:48	6.30	31	22.3	167.3	3.4	25.5	–
51	0:29	6.76	28	6.7	50.3	11.5	86.3	−23
52	–	6.3	>30	–	–	–	–	–
**Range**	**00:29–01:21**	**6.3–6.95**	**8.6–41**	**6.4–22.3**	**48–163.5**	**2.2–21**	**16.5–157**	**−14 to −39**
**Mean**	**0:53**	**6.52**	**26.3**	**14.8**	**111.0**	**7.38**	**55.35**	**−23.7**
**Median**	**0:52**	**6.50**	**30**	**15.3**	**114.8**	**5.69**	**42.68**	**−23.5**

*Note*: –, no data. Bold refer statistical significance.

Autopsy findings are described in Table 4. The majority of cases demonstrated no significant internal and external injuries, though two presented with laryngeal injuries, another two had small tears within the liver and spleen, and in a single case, a laceration from dog bites was noted. The number of individuals exhibiting petechiae was 19 (37%). Pathological findings included cardiac (fibrosis, cardiomegaly, atherosclerosis), hepatic (steatosis, cirrhosis, hepatitis), pulmonary (bleedings, aspiration, bronchitis, pneumonia), and occasional gastrointestinal or renal pathologies across the cases. In the original autopsy reports, the cause of death was typically described as multifactorial, with the most significant factor reported by the forensic pathologist listed in Table 4. Among the 52 cases, positional or traumatic asphyxia was the most common primary cause of death, assigned in 23 cases (44%). This was followed by intoxication with stimulants in 9 cases (17%), cardiovascular‐related causes in 8 cases (15%), exhaustion in 6 cases (12%), asphyxia (unspecified) in 3 cases (6%), and a single case each of capsaicin toxicity, acute alcohol toxicity, and excited delirium (2%). This variability of diagnoses in cases that share so many clinical and forensic features illustrates the absence of consensus within the forensic community, and reflects the challenges in understanding the mechanisms involved in these complex deaths.

**TABLE 4:** Autopsy findings.

Case number	Significant injuries (bruises, abrasions and CPR injuries excluded)	Petechiae	Toxicology		Pathological findings	Primary cause of death provided by the forensic pathologist^a^
			Ethanol	Stimulants		
1	None	Yes	1.9 g/L	2.9 μg/g amphetamine	Cardiac fibrosis, pulmonary bleedings, hepatic steatosis	Cardiovascular‐related
2	None	No	None	3.3 μg/g amphetamine	Cardiac fibrosis, pulmonary, upper airway aspiration, pulmonary bleedings, hepatic steatosis	Intoxication
3	None	Yes	None	1.1 μg/g amphetamine	Hepatic steatosis	Intoxication
4	None	No	None	1.3 μg/g amphetamine	Cardiac fibrosis, chronic hepatitis	Cardiovascular‐related
5	Bilateral forearm fractures.	No	None	None	Hepatatis	Positional asphyxia
6	None	Yes	None	1.5 μg/g amphetamine	None	Asphyxia
7	None	No	None	None	Myocarditis	Cardiovascular‐related
8	None	No	None	None	Aspiration	Positional asphyxia
9	Tears in liver and spleen, 647 mL blood in abdomen.	Yes	None	None	Hepatic steatosis	Exhaustion
10	None	Yes	1.6 g/L	None	Aspiration	Positional asphyxia
11	None	No	None	0.03 μg/g diazepam 0.01 μg/g haloperidol	Cardiac fibrosis, atherosclerosis, chronic nephritis	Cardiovascular‐related
12	Rib fractures. Bite and tear marks in accordance with police dog	No	1.5 g/L	1.1 μg/g amphetamine THC positive	None	Exhaustion
13	None	No	0.7 g/L	3.4 μg/g amphetamine	Cardiomegaly, aspiration	Cardiovascular‐related
14	None	Yes	2.3 g/L	None	Minor gastric ulcers	Asphyxia
15	None	Yes	1.9 g/L	None		Positional asphyxia
16	None	Yes	1.9 g/L	THC positive	Bronchitis	Positional asphyxia
17	None	No	None	2.3 μg/g amphetamine	Cardiac fibrosis, bronchitis, hepatic steatosis	Intoxication
18	None	Yes	None	None	Cardiac fibrosis, atherosclerosis, hepatic steatosis	Exhaustion
19	Small hemorrhage in renal pelvis.	No	1.4 g/L	None	Cardiac fibrosis, atherosclerosis, hepatic steatosis	Positional asphyxia
20	None	No	None	3.9 μg/g amphetamine THC	Hepatic steatosis	Positional asphyxia
21	None	Yes	None	0.07 μg/g alimemazine	Hepatic steatosis	Cardiovascular‐related
22	None	Yes	None	1.7 μg/g amphetamine	Hepatic steatosis	Positional asphyxia
23	None	No	None	0.2 μg/g amphetamine	Previous myocardial infarction, cardiomegaly, atherosclerosis	Cardiovascular‐related
24	None	No	None	1.2 μg/g amphetamine THC positive	Cardiac fibrosis, aspiration	Intoxication
25	None	No	None	2.0 μg/g amphetamine	Bronchitis, hepatic steatosis	Intoxication
26	None	Yes	1.8 g/L	None	Cardiomegaly	Acute alcohol toxicity
27	None	No	None	None	None	Positional asphyxia
28	Rib fractures.	Yes	None	None	None	Positional asphyxia
29	None	Yes	None	0.8 μg/g diazepam 0.0003 μg/g LSD	Atherosclerosis	Positional asphyxia
30	None	Yes	None	None	Cardiomegaly, atherosclerosis, hepatic cirrhosis, minor gastric ulcers	Positional asphyxia
31	None	No	2.5 g/L	0.51 μg/g methylphenidate	Aspiration	Exhaustion
32	None	No	None	1.7 μg/g amphetamine 5.2 μg/g fentanyl	None	Positional asphyxia
33	None	No	None	0.02 μg/g morphine	Pulmonary bleedings, pneumonia	Positional asphyxia
34	None	Yes	None	None	None	Positional asphyxia
35	None	No	None	0.09 μg/g diazepam LSD (not quantified)	Pulmonary edema	Intoxication
36	None	No	None	0.06 μg/g amphetamine. 0.77 μg/g methamphetamine	Cardiomegaly, atherosclerosis	Intoxication
37	Hyoid fracture, vocal cords hemorrhage.	Yes	2.29 g/L	THC positive Testosterone	Cardiomegaly, chronic hepatitis	Asphyxia/strangulation
38	None	No	1.38 g/L	0.57 μg/g cocaine 0.4 μg/g amphetamine	Cardiac fibrosis	Positional asphyxia
39	None	No	Not tested – treated at hospital intensive care unit over a prolonged period prior to death		Cardiomegaly	Exhaustion
40	None	No	None	0.8 μg/g amphetamine	Cardiac fibrosis, atherosclerosis, hepatic steatosis	Cardiovascular‐related
41	None	Yes	1.21 g/L	None	Atherosclerosis, pulmonary bleedings, hepatic steatosis	Positional asphyxia
42	None	Yes	None	4.1 μg/g amphetamine	None	Positional asphyxia
43	None	No	None	0.7 μg/g amphetamine 0.003 μg/g alprazolam	Aspiration, hepatic cirrhosis	Exhaustion
44	None	No	0.24 g/L	0.78 μg/g amphetamine	None	Positional asphyxia
45	Thyroid cartilage upper horn fracture.	No	0.41 g/L	0.36 μg/g amphetamine	Hepatic steatosis	Positional asphyxia
46	None	No	0.27 g/L	9.2 μg/g amphetamine 0.034 μg/g morphine (heroin) 0.034 μg/g methylphenidate	Hepatic steatosis	Intoxication
47	None	No	None	1.0 μg/g amphetamine 0.25 capsaicine (from OC spray)	Pulmonary bleedings, chronic hepatitis, nephrosclerosis, haemorrhagic colitis	Capsaicine intoxication
48	None	No	0.32 g/L	2.8 μg/g amphetamine 0.05 μg/g zopiclone 0.009 μg/g alprazolam	None	Positional asphyxia
49	Small tears in liver and spleen, 500 mL blood in abdomen.	No	None	0.72 μg/g cocaine	None	Intoxication
50	None	No	None	0.95 μg/g cocaine	None	Positional asphyxia
51	None	No	None	0.37 μg/g amphetamine 0.83 μg/g buprenorphine 0.025 μg/g alprazolam	None	Excited delirium
52	None	No	None	0.06 μg/g cocaine	Blod clot in cardiac auricle	Positional asphyxia

*Note*: Cardiomegaly = >500 g men, >400 g women.

^a^ The cause of death was typically multifactorial, with the one listed here representing the primary factor according to the original autopsy report.

Toxicological analysis detected ethanol in 17 cases (33%), with concentrations ranging from 0.21 to 2.29 g/L. Various stimulant substances were identified in 36 cases (69%) across the cohort. Amphetamine was present in 25 cases (49%), with concentrations ranging from 0.06 to 3.9 μg/g, and in 13 cases, it was the only substance detected. Cocaine was found in four cases (8%) at concentrations between 0.06 and 0.95 μg/g. Alprazolam was identified in three cases (6%) (range: 0.003–0.025 μg/g), while methylphenidate was detected in two cases (4%) (0.034 and 0.77 μg/g). Morphine was present in two cases (6%) (0.02 and 0.034 μg/g), and diazepam was detected in two cases (4%) (0.09 and 0.8 μg/g). Lysergic acid diethylamide (LSD) was detected in two cases, though concentrations were only measured in one (0.0003 μg/g). Tetrahydrocannabinol (THC) was identified in five cases (10%), of which one was where THC was the sole substance detected.

In single cases, methamphetamine (0.77 μg/g), fentanyl (5.2 μg/g), zopiclone (0.05 μg/g), haloperidol (0.01 μg/g), and buprenorphine (0.83 μg/g) were detected, all in combination with other substances. In addition, capsaicin (0.25 μg/g), administered via OC spray, was identified in one case and exogenous testosterone was also identified in a separate case.

Neither ethanol nor stimulants were detected in 9 individuals (17%).

## 4 DISCUSSION

The investigation of sudden death in agitated individuals during physical restraint poses substantial challenges at the intersection of medicine and law. Current understanding of the mechanisms leading to death is based on retrospective case data and on inferences from respiratory, circulatory, acid–base and exercise physiology, as well as laboratory studies on healthy volunteers. However, as will be discussed, the value of laboratory studies is inherently limited by ethical and safety constraints. As a result, determining the cause and manner of death requires a careful and multidisciplinary interpretation.

To advance the current body of knowledge, we conducted a nationwide, retrospective study in Sweden spanning a 32‐year period. A total of 52 cases of sudden death during the restraint of agitated individuals were identified. To our knowledge, the present study represents the largest consecutive cohort specifically focused on sudden death during physical restraint and includes findings from emergency clinics and forensic autopsy [27].

The average number of restraint‐related deaths during the study period was 1.58 cases per year (Figure 1), with the national population increasing from 8.7 million in 1992 to 10.6 million in 2024. The average incidence was 0.17 deaths per million inhabitants per year, a figure comparable to that reported in two studies from Ontario, Canada (0.25 and 0.19 deaths per million) [28, 29] and a Dutch study (0.19 deaths per million) [30], but lower than the incidence observed in a study from Maryland, USA (0.58 deaths per million) [31].

The incidence of restraint‐related deaths in Sweden has varied considerably over time (Figure 1). Higher rates were seen in the early 1990s and again after 2015, interspersed by a period of very low incidence between 2000 and 2015. Given the low absolute number of deaths and the complexity of societal factors influencing risk, firm conclusions regarding temporal trends cannot be drawn. However, it is noteworthy that the period of reduced incidence between 2000 and 2015 followed the most widely publicized restraint‐related death in modern Swedish history, that of Osmo Vallo in 1995. In the immediate aftermath of Mr. Vallo's death, instructional content addressing the risks of restraint–asphyxia during the apprehension of agitated individuals was introduced into the Swedish Police Academy curriculum (1997), and a corresponding section was added to the Police Handbook (1998) [1, 32].

The general circumstances leading up to death, observed in the present cohort are consistent with previously reported descriptions of fatalities during the restraint of agitated individuals [8, 33, 34, 35]. Rapid loss of consciousness and absence of palpable pulses occurred during or shortly after a physical struggle with law enforcement or healthcare personnel. In our cohort, 90% of individuals were restrained in the prone position and 67% were overweight or obese. Toxicological screening revealed stimulants in 69% of cases, whereas in 17% of cases, no mind‐altering substances were detected. Common pathological findings included cardiac fibrosis and hepatic steatosis or cirrhosis, which are expected in this population, where a large proportion are substance abusers. Major traumatic lesions were universally absent, and other pathological findings, when present, were not of a nature or severity to make the cause of death immediately apparent in the cases presented.

One of the most striking findings of the present study is the profound acid–base derangement of the ABGs, obtained after hospital arrival while CPR was still in progress (Table 3). Compared with ABGs also sampled during ongoing CPR in other out‐of‐hospital cardiac arrest (OHCA) cohorts, the acidosis identified in the present restraint‐related cohort was generally much more severe. In “non‐restraint” studies, median pH values range from 6.83 to 7.07, median PaCO_2_ values from 8.9 to 11.9 kPa (67–89 mmHg), and median lactate values from 12.4 to 13.6 mmol/L during ongoing CPR [36, 37, 38, 39], with similar values reported as means elsewhere [40, 41, 42, 43, 44, 45]. In contrast, median pH, PaCO_2_, and lactate in the present cohort were 6.50, 15.3 kPa (115 mmHg), and 30 mmol/L, respectively (Table 3).

**FIGURE 2:**
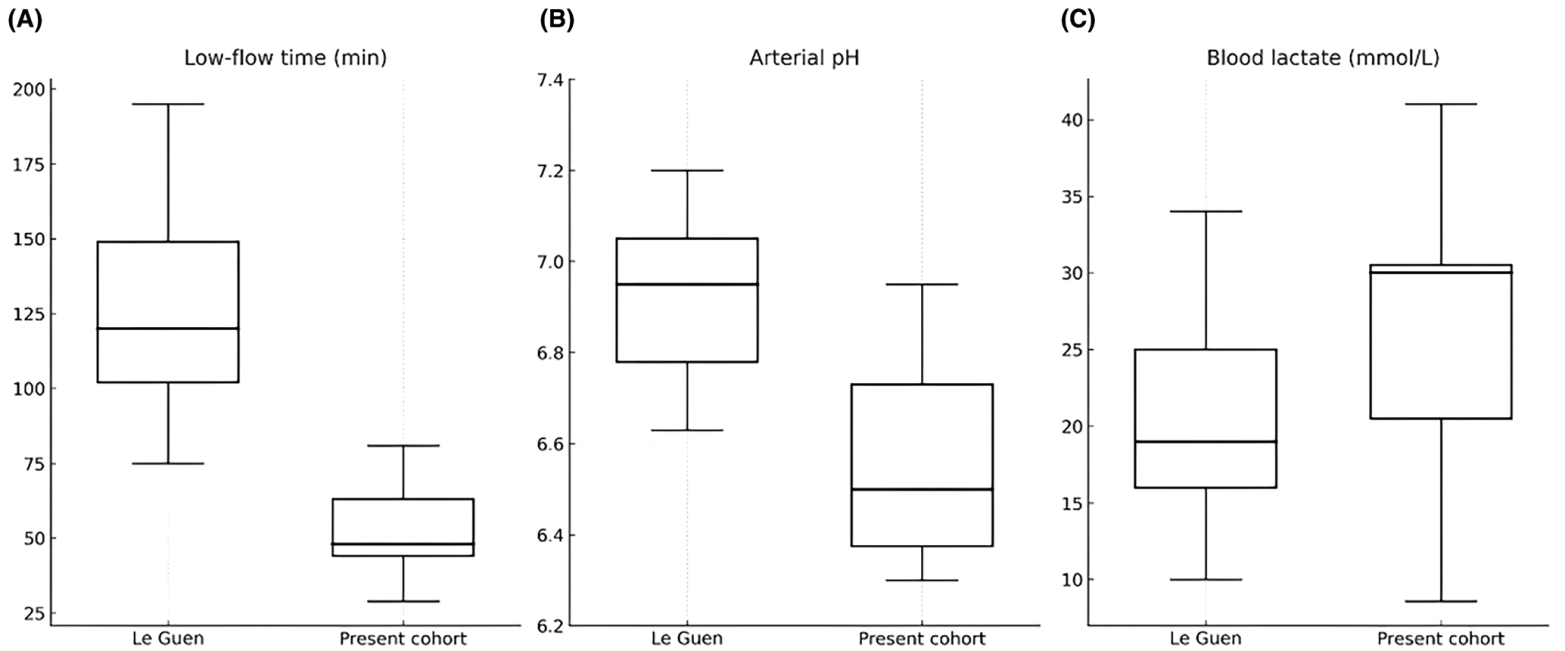
Comparison of (A) low‐flow time (min), (B) arterial pH, and (C) blood lactate (mmol/L) concentrations between the present cohort and data adapted from Le Guen et al. [46]. The low‐flow time of the present cohort is defined as the time from the onset of physical struggle to the time of ABG sampling as the time of hemodynamic arrest could often not be ascertained. Thus, low‐flow time in the present cohort serves as a surrogate measure and likely overestimates the true duration of cardiac arrest. Data from Le Guen et al. [46] were extracted by visual estimation from published box plots (Figure 2) and from Table 1 in the original article. Adapted under CC BY 2.0 (https://creativecommons.org/licenses/by/2.0/). Data are expressed as median (solid line), IQR (boxes), and range (whiskers). For discussion, see the main text.

Perhaps the most relevant comparators are OHCA cohorts in which extracorporeal life support (ECLS) is initiated for treatment‐refractory cardiac arrest. In these studies, it is certain that all ABGs were taken during cardiac arrest, and since implementing ECLS is time consuming, the studies report longer “low‐flow” intervals than do standard OHCA series. “Low‐flow” in these studies refers to the period from collapse until extracorporeal circulation begins, while conventional CPR is in progress. In the present (restraint‐related) cohort, the exact time of cardiac arrest is often impossible to determine, so “low‐flow” is instead defined as the period from the start of the physical altercation leading to collapse, until the time of ABG sampling after hospital arrival. In a study by Le Guen et al. [46], ABGs were obtained immediately before initiation of ECLS in 51 patients after a median low‐flow time of 120 min with acid–base disturbances similar to the non‐ECLS OHCA cohorts [46]. In contrast, median low‐flow time in the present study was 52 minutes, yet the acid–base disturbances were much more pronounced (Figure 2). Two additional OHCA cohorts with patients treated with ECLS report similar values [47, 48].

Consequently, it is reasonable to presume that this difference reflects an acid–base disturbance that developed during the intense physical struggle preceding cardiac arrest in our cohort, a factor that is absent in the non‐restraint OHCA cohorts, where cardiac arrest was most often caused by a primary cardiac event [36, 37, 38, 39, 40, 41, 42, 43, 44, 45, 46, 47, 48].

**FIGURE 3:**
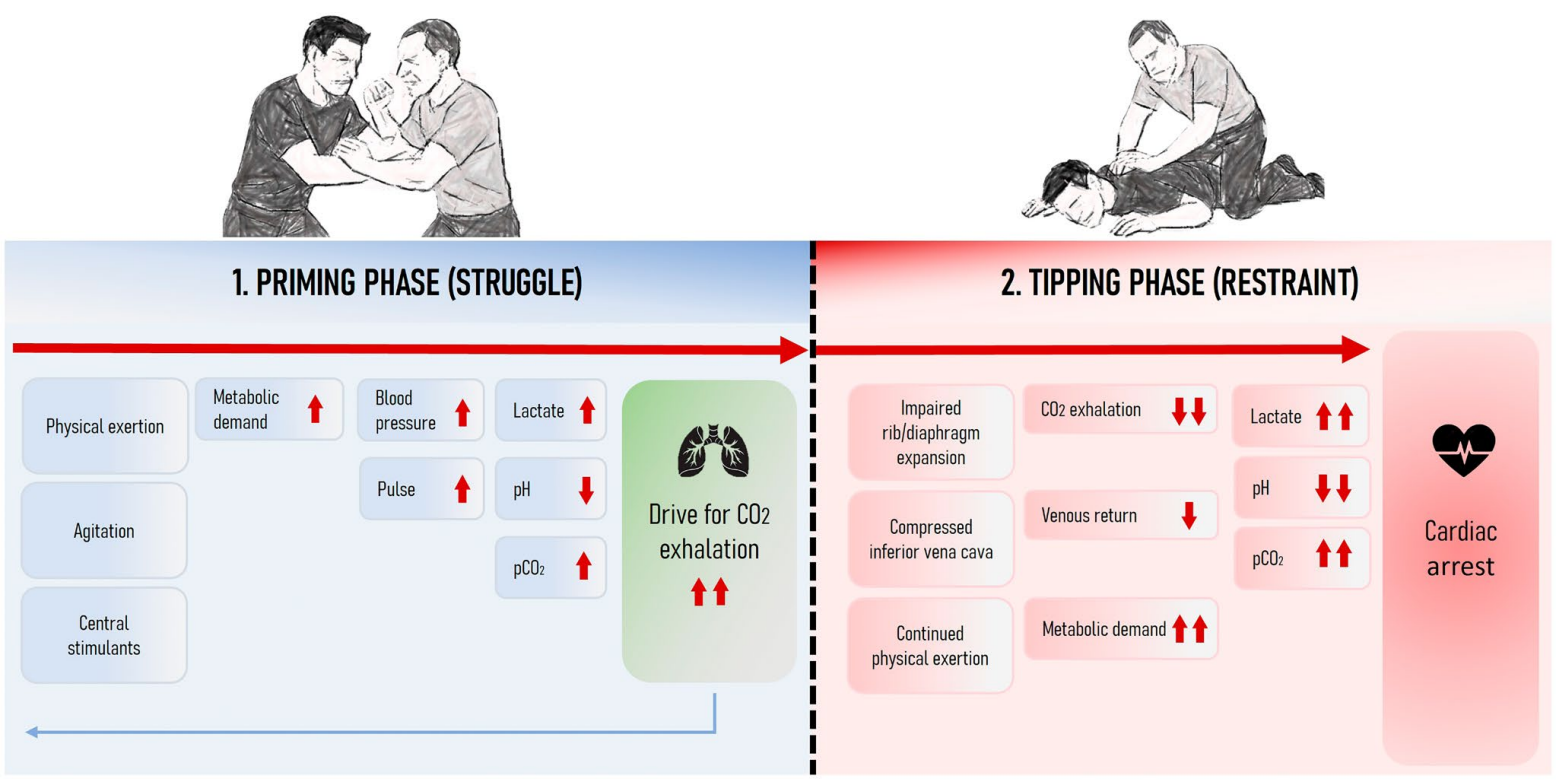
Schematic representation of the proposed two‐phase mechanism leading to *restraint‐related cardiac death during agitation*. In the “priming phase,” agitation and intense physical struggle create pronounced metabolic and physiological stress, with full ventilatory and circulatory effort required to maintain homeostasis. This state of stress may be further exacerbated by central stimulants. Minute ventilation may need to be increased from resting levels of 4–6 L/min to >80–100 L/min to expel excess CO_2_ produced by working muscles and to compensate for the lactic acidosis that develops during maximal exertion. Restraining a highly metabolically stressed individual in prone restraint (as depicted in the figure) will restrict ventilation and diminish venous return that may trigger the “tipping phase”. Reduced ventilation will cause arterial pCO_2_ to rise, exacerbating the established acidemia. Rising alveolar CO_2_ levels can displace alveolar oxygen and cause hypoxia. Delirious individuals typically continue to struggle during the “tipping phase”, further accelerating the negative spiral. The combined effects of worsening acidosis, hypoxia, decreased venous return from compression of the inferior vena cava (in the prone position), and ongoing physiological stress causes a failure cascade that may progress to cardiovascular arrest. Minimizing the time in prone restraint and finding a body position allowing for unrestricted ventilation may alleviate and inhibit the progression of the “tipping phase”, allowing for metabolic recovery and averting the risk of cardiac arrest.

The hypothesis that profound metabolic acidosis develops during exertion and plays a central role in restraint‐related cardiac arrest was first proposed over 25 years ago by Hick et al. [33], and has recently been elaborated on by Steinberg and colleagues [8, 33, 49, 50]. As Hick et al. [33] note, the delirium commonly observed in these cases enables exertion to exceed well beyond normal limits. This can result in a metabolically and physiologically extreme situation, in which full ventilatory and circulatory capacity is required just to maintain homeostasis—a state we refer to as the “priming phase” (Figure 3).

In laboratory conditions with healthy volunteers, mixed venous CO_2_ levels can rise from 6.2 kPa (46.8 mmHg) at rest to 10.4 kPa (78.1 mmHg) during maximal exertion, reflecting the CO_2_ load produced by working muscle [51]. This excess CO_2_ must be expelled through ventilation, which requires minute ventilation to increase dramatically, from resting values of 4–6 L/min to as much as 80–100 L/min. An impaired ability to “blow off CO_2_” also causes a profound “air hunger” and may explain reoccurring exclamations of “I can't breathe” that have been reported during prone restraint [8, 50]. At this point, ventilation must achieve PaCO_2_ levels lower than normal, to compensate for the metabolic acidosis that inevitably arises during such extreme physical exertion.

Under such conditions, any form of interference with breathing [52], in particular prone restraint, will reduce minute ventilation. As a result, the excess CO_2_ present in the mixed venous blood will spill over into the arterial circulation, obviating the respiratory compensation of the metabolic acidosis, further lowering the pH of the already acidotic blood. A reduced ventilation will also lead to alveolar CO_2_ accumulation, which physically displaces oxygen, lowering the alveolar O_2_ tension and impairing O_2_ uptake. The extreme metabolic state induced by the struggle during the “priming phase” thus sets the stage for a subsequent “tipping phase,” where a cascade of physiological failures triggered by the inability to maximally ventilate during restraint, progresses to cardiovascular arrest (Figure 3). It is important to note that while restrained, agitated individuals will often continue exerting themselves, accelerating the progression of the “tipping phase.”

Over the past decades, investigators have published a series of laboratory studies on how different restraint positions affect respiratory and cardiovascular function [53, 54, 55, 56, 57, 58, 59, 60, 61, 62, 63, 64, 65, 66]. In a 2020 synthesis of this work, Vilke [67] reported that the prone position consistently caused restrictive effects on pulmonary function tests and narrows the diameter of the inferior vena cava, signaling impaired venous return, but concluded that none of these findings were clinically relevant [67]. However, some key limitations were acknowledged: the volunteer subjects were fit and healthy, drug‐free and subjected to far less physical and psychological stress than that of agitated individuals encountered in the field. Thus, the protocols may not reflect real‐world risk. Of these limitations, insufficient physical loading is the most consequential. In several protocols, volunteers cycled at about 175 W for 4 min, or until they reached ~85% of their age‐predicted maximal heart rate before being restrained [61, 68]. Such work rates are classified as *moderate intensity* in the context of exercise physiology research and, at this level, blood lactate stays beneath the “onset of blood lactate accumulation” (OBLA) threshold at 3–4 mmol/L [69]. In contrast, truly all‐out exertions that mirror a desperate, life‐and‐death struggle can cause profound acidosis in seconds: a single 30‐s simulated cycle sprint (Wingate‐test) pushes lactate beyond 15 mmol/L^−1^ almost immediately, while a 400‐m sprint and a 2‐km rowing finish can reach 18–30 mmol/L, drive arterial pH into the 6.8 range and bring bicarbonate buffers near zero [70, 71, 72]. It is in this state of depleted physiological reserves that the restrictive effects of prone restraint become capable of tipping the cardiorespiratory system into a failure cascade.

Reproducing such supramaximal exertion in volunteers before placing them in restraint positions would be both unsafe and ethically impermissible. Consequently, the existing volunteer studies, confined to low‐stress conditions, insufficiently mirror the risk in real‐world high‐stress conditions.

Similarly, retrospective reviews of police encounters that ended in prone positioning but recorded no fatalities, shed little light on the risk pathway outlined above [15, 73, 74]. Prone restraint by itself is not intrinsically lethal, and most police altercations do not involve delirious and agitated subjects exerting themselves beyond their physiological limits. The same distinction explains why no deaths are reported in grappling sports such as wrestling or judo: although athletes can finish elite matches with blood‐lactate concentrations of 10–15 mmol/L, they remain fully conscious and the hold is released the moment a competitor yields or the referee stops the bout, conditions drastically different from the chaotic restraint of an agitated individual [75, 76].

As in previous studies [30, 31, 77], stimulants were detected in a high proportion of restraint‐related deaths in the present series. Amphetamine was the most frequently identified substance, present in 49% of cases, most likely reflecting its high prevalence in Sweden. Cocaine, the most commonly occurring stimulant in previous publications [28, 30, 78], was found in only 4 cases (8%). A primary pharmacological effect shared by all stimulants is the elevation of central dopamine levels through reuptake inhibition or direct release. Stimulant use, particularly in the context of binge consumption, is strongly associated with agitation and psychotic symptoms [79, 80, 81]. While the occurrence of stimulants in restraint‐related deaths is well documented, possible causal mechanisms remain the subject of controversy [82, 83]. Of particular relevance to the present context, though not previously emphasized, stimulants have been shown to enable both laboratory animals and human research subjects to override a physiological mechanism known as “central fatigue” [84]. This mechanism normally serves to limit physical exertion when rising body temperatures threaten homeostasis. Thus, stimulants may play a direct role in enabling supramaximal physical exertion to occur during the priming phase [84]. As already noted, however, a substantial number of cases in our series demonstrated no evidence of stimulant use, suggesting that pharmacologic inhibition of central fatigue is not a necessary precondition.

The issue of arrest‐related deaths has been the subject of intense and often acrimonious debate for decades. For the sake of argument, we will here simplify the two polarized positions of this debate as follows:

Argument 1: *Restraint has nothing to do with the deaths of these individuals. Death is caused by a condition called ExD syndrome*.

Argument 2: *Restrained individuals are killed by the police. ExD syndrome is a fictional diagnosis created to excuse police brutality*.

In this simplified form, it is clear that both positions are flawed. Argument 1 fails to consider the exceptional metabolic state that is induced during “the priming phase.” From this physiological starting point, most forms of restraint will have an adverse effect on homeostasis, and the maximum restraint position (or “hobble position”) is in no way “physiologically neutral,” as has been claimed [85].

Remarkably, Argument 2 fails in the same way. The dismissal of ExD as a fictitious diagnosis overlooks the exceptional metabolic state brought on by the “priming phase.” This state is not the result of ordinary physical struggle, but appears to occur almost exclusively in individuals experiencing delirium with severe agitation. While uncommon, this exceptional state of agitation is a precondition for the physiological stresses of the “priming phase” to occur as argued above. Without delirium, there is no “priming phase”; and without the ‘priming phase,’ routine restraint techniques employed will not trigger the “tipping phase.”

The present study does not prove that restraint‐related deaths follow the two‐step sequence of a “priming phase” followed by a “tipping phase” triggered by restraint, as depicted in Figure 3, and conclusive proof may never be attainable. In some cases, pre‐existing pathologies may have also contributed to the death. Nevertheless, three consistent features argue for a common mechanism:

1. Circumstances: every case involved a high‐intensity struggle immediately followed by some kind of restraint; in 90% of cases, a prone position occurred that is known to restrict ventilatory function.
2. Risk factors: stimulant exposure and obesity reoccur.
3. Physiology: the peri‐arrest ABG was profoundly deranged, indicating a severe acidotic state.

Taken together, these observations point to cardiovascular collapse during restraint, superimposed on the extreme metabolic state generated during the “priming phase,” as the most coherent explanation.

The persistent controversy over sudden death during restraint underscores that the underlying mechanism remains poorly understood. If every professional group involved in apprehending agitated individuals recognized the inherent risk of fatal collapse, it may be contended that at least *some* of the unexpected deaths could be prevented.

It should also be emphasized that the authors of this article do not dispute the importance of restraint in protecting both the individuals applying it and the broader community from further harm. Certain situations will require individuals to be restrained, even if this may be conducted in a manner that restricts chest expansion and breathing, such as the use of a prone position. Nevertheless, whether it is labeled agitated delirium, ExD, or hyperactive delirium with agitation, the condition must be treated as a high‐risk medical emergency. Prone restraint in such circumstances is hazardous; it should be avoided where possible and, when unavoidable, applied for the shortest feasible time. Targeted education and the development of best‐practice training for managing this complex emergency are essential. We echo the call of Bivens et al. [50] for a dialogue between emergency physicians, paramedics, and police on the appropriate management of restrained individuals who report, “I can't breathe.”

### 4.1 Limitations of the study

This study is retrospective in design, which inherently limits its evidentiary strength and introduces potential risks of selection and information bias. Retrospective analyses rely on existing records that may be inconsistent or subject to interpretative variability over time. This is an important aspect to consider since the study spans a 32‐year period during which autopsy protocols, diagnostic technologies, and clinical practices have evolved. Variability in methods and the fact that different forensic pathologists conducted autopsies may have introduced inconsistency in data collection and interpretation.

Second, the dataset is incomplete. ABG analyses were only available from 2005 onward, and even within the available subset, certain key parameters are missing in certain cases. This limits the ability to conduct uniform comparisons across cases and time periods. Another limitation involving the ABG results is its timing. While ABG samples were obtained within an average of 53 min following struggle onset, the exact timing of cardiac arrest is difficult to determine.

## 5 CONCLUSIONS

This nationwide, 32‐year retrospective study identified 52 cases of sudden death during the restraint of agitated individuals in Sweden. In 90% of cases, death occurred following restraint in the prone position. Stimulants were detected in 69% of cases, and two‐thirds of the deceased were either overweight or obese. In 15 cases, ABG parameters from the peri‐arrest period were available. ABG values demonstrate very high degrees of combined metabolic (lactate) and respiratory acidosis, with a median pH of 6.5. Based on the results of the present study, we propose a two‐phase model explaining the causal mechanism of *restraint‐related cardiac arrest during agitation*, that builds on that of Steinberg et al. [49]. The first, “priming phase” occurs when an intense physical struggle induces a metabolic state that demands maximal respiratory and cardiovascular function to maintain homeostasis. If physical restraint follows that restricts chest expansion and the ability to ventilate CO_2_, particularly the prone position, the respiratory and cardiovascular systems can no longer compensate, triggering the second “tipping phase” that may progress to cardiovascular collapse and ultimately, death.

## FUNDING INFORMATION

This work was fully supported by the Swedish National Board of Forensic Medicine.

## CONFLICT OF INTEREST STATEMENT

The authors certify that they have no affiliations with or involvement in any organization or entity with any financial interest (such as honoraria; educational grants; participation in speakers' bureaus; membership, employment, consultancies, stock ownership, or other equity interest; and expert testimony or patent‐licensing arrangements), or non‐financial interest (such as personal or professional relationships, affiliations, knowledge or beliefs) in the subject matter or materials discussed in this manuscript.

## Data Availability

The data that support the findings of this study are available on request from the corresponding author. The data are not publicly available due to privacy or ethical restrictions.
